# St. John Long

**Published:** 1831-01-01

**Authors:** 


### XLVII.

### St. John Long.

Another murder, softened down by courtesy into the mild designation of manslaughter, has *come out*—for the two on record, do not form a tenth of those which are, as yet, covered by the earth! We are so sick of the scene, that we shall not harrow up our own or our readers' feelings upon this occasion, by many reflections on the credulity and the *cruelty* of man, who is, on more than one side of the channel, a mixture of the monkey and the tiger! Again we shall be edified with the sight of Marchionesses and their pretty daughters ogling from the bench that interesting *creature* the *gentleman-prisoner* at the bar, while the seat of justice shall over-flow with heaven-born mercy, and tears of pity shall roll from the eyes of Minos and Radamanthus to wash away the *errors* of *judgment* to which all humanity is liable, and into which the gentleman at the bar *might* perhaps have innocently fallen, though even that is not probable. Again we shall be obliged to listen to the stuff collected from the ambitious little misses of Bermondsey, Camberwell, and Lambeth, who longed to disrobe and shew their swelling bosoms, in company with Marchionesses. Again we shall see some precious specimens of aristocratic intellect, delivered from the right and left of the throne of justice, while rude science and vulgar knowledge must descend to the witness-box, to deliver truths unpleasant to noble ears, and uncongenial with legal subtleties! Again we shall be condemned to hear the doctrine laid down, that the evidence of a Marquess, a Marchioness, or a Lord touching the skill and attention of a medical practitioner, must be conclusive on those points, and outweigh the proofs of facts adduced by ten thousand physicians, surgeons, or other plebeian witnesses. Again we shall be reminded that medical men who, during a long life, may have gone on in the accumulation of knowledge and reputation, are precisely on a par with the tinker or the blacksmith, when suspicion of misconduct occurs, or when the merits of practice come to be judicially investigated! This is a doctrine which evidently presumes that a Sir Astley Cooper is just as likely to destroy the life of a fellow-creature by *ignorance*, as a St. John Long, who could not tell the sciatic nerve from the sartorius muscle, for the best of all reasons, that he never saw either the one or the other. We acknowledge, indeed, that medical men must be held liable to criminal actions, as well as quacks—because they may be ignorant notwithstanding their licence to practise—and they may be, and no doubt sometimes are, so negligent as to occasion death. So far they undoubtedly stand upon equal terms with the quack, as far as *liability* to criminal action is concerned. But in all other respects, they ought to be viewed by the bench and by the jury in a very different light. When an educated and licensed man is arraigned for mala praxis, the *presumption* ought to be that mala praxis is improbable, till its existence has been proved beyond a doubt: whereas the *presumption of mala praxis* ought to lie, in reason, against the quack, till evidence has shown that it did not obtain. In short, *mala praxis* should never be *presumed* in the case of the qualified, nor *bona praxis* in that of the charlatan. If such happen in the latter case, it is more by accident than skill.

But we shall drop the subject. It would appear to us, and it seems to be also the opinion of those who are "learned in the laws," that Providence permits certain ways and means for thinning the population of countries, not very consistent with the ideas which we usually attach to infinite wisdom and justice. Plagues and pestilences have been let loose upon all nations, from time to time, as their population became redundant. When Columbus opened out a wide field for increasing the means of subsistence, and consequently for extending the population, syphilis arose, de novo, to check the principle of fecundity—when the East was over-run by our countrymen in search of pagodas, Cholera came forth to thin their ranks—when the

Huns can now no longer swarm over the Alps, to bask in the sunny glades of Italy, the cholera, or some NOVA PESTIS is come out to pay them a visit, and diminish their numbers. So, perhaps, when the *ennui* of certain branches of our aristocracy tired out the patience of our regular physicians and surgeons, and exhausted the shops of æther and hartshorn, Providence has sent them a ST. JOHN LONG to brand their backs with marks of ignorance more conspicuous even than his own, and thus to give a stimulus to the lagging current of their listless existence, and excite some feeble *sensations* in beings who are utterly incapable of *perception* or *reflection!* Such may be the will of Providence — and no doubt we shall hear this line of argument amply illustrated from the bench in favour of the "gentleman at the bar." The verdict of the jury will be of little avail. The aristocracy and their idiotic imitators are not to be deprived of the only stimulation which their decaying carcases are susceptible of, because it drives sound constitutions into fever, inflammation, and death.

---

P.S. Since the above lines were written, the public is informed that certain circumstances prevent the "gentleman at the bar" from paying his devoirs to the Marchioness of Ormond, Justice Park, and other of his great friends, during the present foggy weather. He is positively to favour the public with a second appearance at the Old Bailey in the course of the Spring!

---

